# Toxicities of Immune Checkpoint Inhibitors: Itis-Ending Adverse Reactions and More

**DOI:** 10.7759/cureus.6935

**Published:** 2020-02-10

**Authors:** Moataz Ellithi, Radowan Elnair, Guy Vin Chang, Mohamed A Abdallah

**Affiliations:** 1 Internal Medicine, University of South Dakota Sanford School of Medicine, Sioux Falls, USA; 2 Hematology and Oncology, University of Nebraska, Omaha, USA; 3 Internal Medicine, University of South Dakota, Sioux falls, USA; 4 Internal Medicine, University of South Dakota, Sioux Falls, USA

**Keywords:** immune checkpoint inhibitors, adverse reactions, immune mediated side effects, nivolumab, ipilimumab

## Abstract

Immune checkpoint inhibitors (ICI) have revolutionized the treatment of cancer worldwide. Not long ago, before the introduction of ICIs, many cancers incurred a grave prognosis on patients due to the lack of effective therapies. For instance, patients with malignant melanoma survive longer and experience a better quality of life than ever before due to agents such as nivolumab and ipilimumab. Nevertheless, toxicities associated with the use of ICIs have been increasingly recognized in clinical trials as well as oncology practice. The widespread usage of ICIs and the expected addition of newer ICIs to the arsenal of medications to fight cancer raise awareness of the potential toxicities of these medications. Once these toxicities develop, immunosuppression with or without withholding immunotherapy is the standard of care. Because the long-term adverse effects of these toxicities and the impact of stopping therapy on survival are not well characterized, a joint decision by both the oncologist and the patient should be carried out if stopping therapy is being considered. Nevertheless, long-term data is necessary to guide such decisions. In this article, we will discuss common ICI’s immune-related adverse events with a simplified approach to recognizing and managing these events.

## Introduction and background

Immune checkpoint inhibitors (ICI) are monoclonal antibodies that activate the immune system by inhibiting agents that downregulate the function of T-cells. ICIs block several targets, such as cytotoxic T lymphocyte antigen 4 (CTLA-4), programmed death 1 (PD-1), and programmed death ligand1 (PD-L1), which in turn disinhibit proliferation of antitumor T-cells [1]. ICIs, alone or in combinations, have expressed positive clinical efficacy and demonstrated survival benefit against many cancer types in many randomized clinical trials. Agents such as ipilimumab (CTLA-4 inhibitor), nivolumab, pembrolizumab (PD-1 blockers), and durvalumab (PD-L1 blocker) are approved by the Food and Drug Administration (FDA) to treat many tumor types, including melanoma, renal cell carcinoma (RCC), urothelial cancers, non-small cell lung cancer (NSCLC), small cell lung cancer, Hodgkin’s lymphoma (HL), hepatocellular carcinoma (HCC), colorectal cancer, and squamous cell carcinoma of head and neck, among many others [1-3]. While both ICIs and chemotherapy are used in the treatment of malignancies, immunotherapy is quite different from conventional chemotherapy in terms of mechanism of action, side effect profile, and management approach of side effects. One main difference is that toxicities attributed to chemotherapy are secondary to immunosuppression, while on the other hand, toxicities related to ICIs are largely from immune activation.

The development of “immune-related adverse events” (irAEs) is thought to be secondary to the enhancement of the immune system activity against tumor cells. The resultant autoimmunity and inflammatory reaction against host-tissue mediated by T-cell, antibodies, and cytokines largely responsible for such toxicities [1]. Exacerbation of the existing organ inflammation, genetic predisposition and antigenic resemblance between malignant and host cells are some of the proposed underlying mechanisms for the development of autoimmunity [3]. Incidence and severity of irAEs vary depending on many risk factors: host-related factors, the ICIs used, its dose and mechanism of action, the combination of more than one ICI agent, and the pre-existent autoimmune diseases in the affected subject [1,3].

The significance of irAEs stems from the fact that up to half of the patients who receive ICIs experience these side effects [4]. In fact, many studies have speculated that the incidence of clinically significant immune-related adverse reactions might have been under-reported in clinical trials, and toxicities are likely more frequent and more severe in the clinical setting. For example, in a study of 64 patients who were treated with nivolumab and ipilimumab for melanoma, 91% of patients had clinically significant reactions and about 72% of patients required at least a dose of systemic steroids over a period of three months. About a third of the patients in that study required temporal or permanent discontinuation of therapy [5]. The discrepancy in reporting irAEs can be due to the delayed nature of some of these reactions, using a combination therapy of two or more ICIs, or due to the fact that clinical trials typically recruit healthier patients with few comorbidities in comparison to patients seen in clinical practice.

While irAEs affect any organ system, dermatologic, gastrointestinal, pulmonary, endocrinological, hematologic, and musculoskeletal adverse events are among the most commonly reported [1]. The irAEs from CTLA-4 inhibitors, when compared to PD-1/PD-L-1 blockers, are usually more common, more severe, and exhibit a dose-dependent pattern [1-3]. Interestingly, there is growing evidence that supports a correlation between the development of irAEs and overall response to ICIs. Two studies have shown that skin toxicities, including rash and vitiligo, were associated with a better overall survival benefit in patients with advanced melanoma receiving ICIs [3,6]. However, no significant survival differences were seen with other irAEs in one of these studies [3]. In another study, the objective response rate (ORR) was significantly higher in patients who experienced treatment-related select irAEs of any grade compared with those who did not [7]. Further studies are needed to further characterize the correlation between those adverse effects and overall outcome.

The Common Terminology Criteria for Adverse Events (CTCAE version 5) was designed initially to classify toxicities from chemotherapy, but it is largely used to grade toxicities resulting from immunotherapy as well [8]. The criteria grade toxicities from grade 1 (G1) to grade 4 (G4), with one being the mildest and four being the most severe form. In general, grade 1 toxicities are mild and do not indicate stopping treatment, whereas grade 3 (G3) or G4 toxicities usually require stopping treatment and/or hospitalization.

## Review

Gastrointestinal immune-related adverse effects

Colitis is one of the most common irAEs, and the incidence of colitis ranges between 5% for PD-1 and PD-L1 and 25% for CTLA-4 [2]. Patients typically present with watery diarrhea, abdominal pain or gastrointestinal bleeding [8]. Before considering a diagnosis of immune-mediated colitis, exclusion of other etiologies of acute diarrhea is necessary, especially infectious causes [8]. Stool testing for lactoferrin and calprotectin can be used to differentiate functional from inflammatory diarrhea and in monitoring disease activity. Also, screening laboratories for human immunodeficiency virus (HIV), hepatitis A and B, and blood quantiferon testing for tuberculosis should be obtained in patients with high-risk features in whom infliximab therapy would be started [2,8]. Classification to G1-G4 is based on the frequency of bowel movements, presence of life-threatening complications, the need for hospitalization or impairment of functional status [8]. Patients with G1 are managed conservatively with hydration, antidiarrheal therapy, and withholding ICIs temporarily. For patients with grade 2 (G2) or more, treatment with corticosteroids (prednisone 1-2 mg/kg/day or equivalent, or 1-2 mg/kg/day methylprednisolone or equivalent) for G4 with tapering over four to six weeks, and consultation with gastroenterology for evaluation for endoscopy is recommended [8]. Esophagogastroduodenoscopy and/or colonoscopy is useful for diagnosis, exclusion of other etiologies and in the stratification of patients who might be candidates for early infliximab therapy. Colonic biopsy showing intraepithelial neutrophils and increased crypt/gland apoptosis support the diagnosis of immune-mediated colitis. Patients with a suggestive clinical picture for colitis should be treated promptly with high-dose steroids while awaiting colonoscopy [2]. In patients non-responsive to steroids in three to five days, infliximab or vedolizumab therapy (for diarrhea refractory to infliximab or with contraindications to tumor necrosis factor-alpha (TNF-α) inhibitors) is recommended [8]. In patients with G2 or G3, consideration of stopping CTLA-4 is recommended and permanent discontinuation of ICIs should be considered in patients with G4 toxicity. PD-1, PD-L1 may be restarted if patients can recover to G1 or less [8].

Hepatitis is suspected in the elevation of liver enzymes and/or bilirubin levels. Elevation of liver enzymes occurs in 1% of patients on anti-PD-1/PD-L1 and in 10 % on anti-CTLA-4 agents [2]. Patients on ICIs require frequent monitoring of liver enzymes while on immunotherapy [8-9]. Patients on ICIs with evidence of elevated aspartate transaminase (AST), alanine aminotransferase (ALT), and or total bilirubin should be graded based on the level of elevation. Liver biopsy demonstrating immune cell infiltration of the liver can aid in the diagnosis but is not routinely performed [9]. G1 is defined by AST/ALT elevation up to three upper limits of normal and/or total bilirubin more than one and a half upper limits of normal [8]. Close monitoring of liver enzymes and supportive care is recommended. However, in patients with G2 or more, avoiding hepatotoxins, frequent monitoring of liver enzymes, and working up other causes of elevated liver enzymes, in addition to withholding ICI is recommended [8]. Oral or intravenous corticosteroids with four to six weeks taper are recommended depending on the severity of elevation of liver enzymes and/or the presence of symptoms. Infliximab is not recommended, given the potential risk for idiosyncratic liver failure [8]. In patients with no response in three to five days, azathioprine or mycophenolate mofetil and hepatology consultation should be considered. Permanent discontinuation is recommended for G3 and G4 toxicities [8].

Pulmonary immune-related adverse effects

Affected patients usually present with pneumonitis. Patients with pneumonitis attributed to ICIs typically have evidence of diffuse inflammation of the lung parenchyma on computerized tomography (CT) imaging of the chest [1,2,8]. Exclusion of common differential diagnoses of pneumonitis, likely pulmonary embolism and pneumonia, is necessary. Regardless of toxicity grade, ICIs should be held and CT imaging (or spirometry if initially done) should be repeated in three or four weeks to monitor progression. Monitoring with history, physical examination, and pulse oximetry are essential as well. Prednisone 1-2 mg/kg/d with taper by 5-10 mg/week over four to six weeks, bronchoscopy with bronchoalveolar lavage (BAL) and empirical antibiotics are recommended for G4. If there is radiologic evidence of improvement or resolution to G1 or less, ICI can be resumed [8]. In G3/G4, permanent discontinuation of ICIs, intravenous (IV) corticosteroids, empirical antibiotics; and if there is no improvement after 48 hours, infliximab, mycophenolate mofetil, intravenous immunoglobulin (IVIG), or cyclophosphamide may be added. Pulmonary and infectious disease consultation with or without bronchoscopy (if not done initially) might be considered [8].

Rheumatic immune-related adverse effects

The largest single-center cohort of rheumatic immune-related adverse effects (Rh-irAEs) studied to date is a retrospective analysis done on a cohort of 1293 patients who received ICIs. Sixty-one patients developed Rh-irAEs with a prevalence of 4.7%, and 14 patients had preexisting documented autoimmune diseases [10]. While this study suggests that Rh-irAEs are not very common, it also predicts that they may be underestimated due to inconsistent reporting and the lack of defined characterization of Rh-irAEs [10]. The most common Rh-irAEs found in this study were inflammatory arthritides, with a prevalence of 2% of all patients who received ICI, with an average time to symptoms onset of four to eight weeks from initiation of ICI therapy [10]. More than 90% of the patients with inflammatory arthritis continued ICI therapy while undergoing treatment for Rh-irAEs [10]. A previous study of patients with rheumatoid arthritis (RA) showed an increased expression of PD-1 in synovial tissue, compared to control, suggesting one potential mechanism of how ICI may induce disease flares or unmask preclinical RA [11]. Contrary to the theoretical concern that immunosuppression may suppress antitumor response, a retrospective study of overall survival and time to treatment failure in patients receiving glucocorticoids or TNF-α inhibitors showed no difference compared with controls [12]. This result was similar to what was shown in another study that looked at the safety of nivolumab monotherapy in patients with advanced melanoma, as the use of immunomodulatory did not affect the objective response rate (ORR) [7]. For other rheumatological irAEs, myopathy occurred in about 0.8% of patients' cohort, and all affected patients presented with myalgia and weakness [10]. Half of the myopathies had bulbar myopathy, and up to 40% had concomitant myocarditis [10]. Vasculitis, polymyalgia rheumatica (PMR)-like syndrome, connective tissue disease (CTD), or flaring of a preexisting rheumatic disease were observed in few patients and seemed to be rarer than inflammatory arthritis and myopathy [10]. Among the above-mentioned Rh-irAEs, myopathy was the most severe Rh-irAE encountered and required permanent discontinuation of ICI therapy in most patients, but morbidity and mortality were particularly high in those who had concomitant myocarditis [10]. In addition to Rh-irAEs, the safety of ICIs is less clear in these with preexisting autoimmune disease who are eligible for ICI therapy, as they were excluded in most of the clinical trials. Six patients out of the 14 patients with preexisting autoimmune disease included in one study had disease flares, with hospitalization required only in one patient [10]. Although several retrospective studies have suggested that most of those patients can be safely treated with ICIs, the clinical practice remains variable as no prospective trials have investigated the safety profile of ICIs in this cohort of patients [1].

Endocrine immune-related adverse effects

Retrospective analysis data done on patients who received ipilimumab in a single comprehensive cancer center found that the most commonly encountered endocrinological irAEs were hypophysitis and thyroiditis, with an overall incidence of 8% and 6% respectively [13]. Investigators have suggested that immune-related hypophysitis is likely is caused by complement activation and a type II hypersensitivity reaction to ectopic CTLA-4 protein co-expressed on pituitary cells [14]. Patients should be educated about symptoms of ICI-related hypophysitis and routine monitoring with early-morning adrenocorticotropic hormone (ACTH) and cortisol levels at baseline and during treatment should be considered [15]. Initial management of hypophysitis is through the replacement of deficient hormones, specifically steroids and thyroxin hormones. High-dose corticosteroids can be used for those with a critical illness, significant hyponatremia, or visual abnormalities. It’s important to note that glucocorticoid deficiency should be first treated to avoid any potential adrenal crisis with initial thyroxin replacement [16]. The mechanism of thyroiditis is not clear, although some investigators hypothesized that polymorphic variants in the PD-1 gene in certain individuals can predispose to an increased risk of endocrine dysfunction with PD-1 blockers [17]. Monitoring for progression to hypothyroidism is essential with regular follow up of symptoms, signs, and thyroid-stimulating hormone (TSH) levels [8]. Levothyroxine hormone replacement should be initiated if overt hypothyroidism develops [18]. Classification of thyroid disorders-associated toxicities to G1 to G4 according to the level of TSH elevation, presence/severity of symptoms and functional assessment guide management and stratification of patients [8]. Endocrinology consultation and IV supplementation of thyroid therapy might be needed for G3/G4 in addition to discontinuation of ICI [8]. Type 1 diabetes mellitus has been previously reported in association with ICI use, which necessitates careful monitoring of patients for elevated levels of blood sugar [16].

Neurologic immune-related adverse effects

Neurological irAEs are rare with an incidence of <1% of patients treated with ICIs [2]. However, severe neurotoxicity includes Guillain-Barre syndrome, myasthenia gravis, and encephalitis have been reported [2]. The occurrence of such toxicities requires prompt disease-specific management, along with further workup with imaging with or without lumbar puncture to rule out alternative etiologies, including cancer progression or infections [19]. Myasthenia gravis and encephalitis were more associated with anti-PD-1 whereas other neurologic AEs were associated with anti-CTLA-4. Myasthenia gravis was characterized by high fatality rates (~ 20%), early-onset (median 29 days) and frequent concurrent myocarditis and myositis [20].

Dermatologic immune-related adverse effects

Skin toxicities occur in up to 30% of patients treated with ICIs and may range in clinical presentation from pruritus and acneiform rash to toxic epidermal necrolysis [2,8]. Blisters or mucosal involvement are rare (<1%) but suggest a bullous disorder or Stevens-Johnson syndrome, and require prompt dermatology evaluation and high-dose steroids [8]. Full skin and mucosal examination are paramount in every follow-up visit while on ICIs. Mild reactions can be effectively managed with topical steroids and antihistamines. Persistent or more severe cases may require high-dose systemic steroids [10].

## Conclusions

Due to the lack of prospective clinical trials to guide the management of immune-related adverse effects of immunotherapy, expert opinions based on observational studies have driven the development of standardized guidelines for the management of these side effects. There are available clinical practice guidelines to help clinicians and patients decide on the management plan, and a multidisciplinary team approach is recommended. In general, grade mild toxicities can be managed by the temporal cessation of the offending medication and therapy can be restarted once toxicities resolve. Moderate and severe toxicities may require the administration of systemic steroid treatment and/or another immunosuppressive regimen in addition to the permanent cessation of immunotherapy for the majority of severe toxicities. Endocrinological adverse reactions can be managed with hormone replacement, and immunotherapy can be continued. Other immunosuppressant agents, such as infliximab and mycophenolate mofetil, are beneficial in patients refractory to steroids.
